# Supplementary material for: Green Synthesis of Selenium Nanoparticles by Grape Seed Extract Synergized with Ascorbic Acid: Preparation Optimization, Structural Characterization, and Functional Activity
Source: Foods. 2025 Aug 27;14(17):3002. doi: 10.3390/foods14173002 (PMC12427814; doi:10.3390/foods14173002)

Table S1. Response surface experiment design.

Factors	Level		
	-1	0	1
Concentration of ascorbic acid (mM)	4	5	6
Concentration of GSE (g/mL)	350	500	650
Reaction time (h)	1	1.5	2
Reaction temperature (°C)	35	45	55

Table S2. Box–Behnken design and experimental responses of the value Y.

Run	X_1	X_2	X_3	X_4	PDI
1	4	650	1.5	45	0.194
2	4	350	1.5	45	0.211
3	4	500	1.5	55	0.189
4	5	650	2	45	0.196
5	4	500	1.5	35	0.198
6	5	500	1	55	0.207
7	5	500	1.5	45	0.157
8	5	350	2	45	0.215
9	6	500	1	45	0.222
10	5	500	2	35	0.191
11	5	350	1.5	35	0.201
12	6	500	1.5	55	0.214
13	6	350	1.5	45	0.216
14	5	500	1	35	0.217
15	5	650	1	45	0.207
16	5	350	1	45	0.230
17	5	350	1.5	55	0.205
18	5	650	1.5	55	0.204
19	5	500	2	55	0.221
20	4	500	2	45	0.200
21	5	500	1.5	45	0.162
22	6	650	1.5	45	0.203
23	4	500	1	45	0.219
24	6	500	2	45	0.225
25	5	650	1.5	35	0.199
26	6	500	1.5	35	0.189
27	5	500	1.5	45	0.159

Table S3. Analysis of variance of response surface regression equation.

Source	Sum of Squares	df	Mean Square	F-value	P-value	
Model	0.0089	14	0.0006	20.76	< 0.0001	significant
X_1	0.0003	1	0.0003	9.12	0.0107	*
X_2	0.0005	1	0.0005	15.25	0.0021	**
X_3	0.0002	1	0.0002	7.9	0.0157	*
X_4	0.0002	1	0.0002	5.49	0.0372	*
X_1*X_2	4.00E-06	1	4.00E-06	0.1301	0.7246	
X_1*X_3	0.0001	1	0.0001	3.94	0.0706	
X_1*X_4	0.0003	1	0.0003	9.4	0.0098	**
X_2*X_3	4.00E-06	1	4.00E-06	0.1301	0.7246	
X_2*X_4	2.50E-07	1	2.50E-07	0.0081	0.9296	
X_3*X_4	0.0004	1	0.0004	13.01	0.0036	**
X_1^2	0.0029	1	0.0029	92.77	< 0.0001	**
X_2^2	0.0029	1	0.0029	93.78	< 0.0001	**
X_3^2	0.0054	1	0.0054	176.26	< 0.0001	**
X_4^2	0.0016	1	0.0016	53.13	< 0.0001	**
Residual	0.0004	12	0			
Lack of Fit	0.0004	10	0	5.62	0.1603	not significant
Pure Error	0	2	6.33E-06			
Cor Total	0.0093	26				

Notes: $p < 0.01$ indicates highly significant differences (**), while $p < 0.05$ indicates significant differences (*). "Not significant" denotes lack of statistical significance ($p > 0.05$).

Figure S1. Effects of single-factor optimization experiments on particle size, PDI, and UV–Vis absorption characteristics of GSE-SeNPs. (a, b) Effect of different Vc concentrations; (c, d) effect of different GSE concentrations; (e, f) effect of different reaction times; (g, h) effect of different reaction temperatures. In subfigures a, b, e, and g, bar charts show particle size and PDI of GSE-SeNPs under varying conditions. In subfigures b, d, f, and h, corresponding UV–Vis absorption spectra are presented. In particle size and PDI graphs, different uppercase letters indicate significant differences ($p < 0.05$) in particle size, and different lowercase letters (a–d) indicate significant differences ($p < 0.05$) in PDI. Vertical bars represent ± SD ($p < 0.05$).

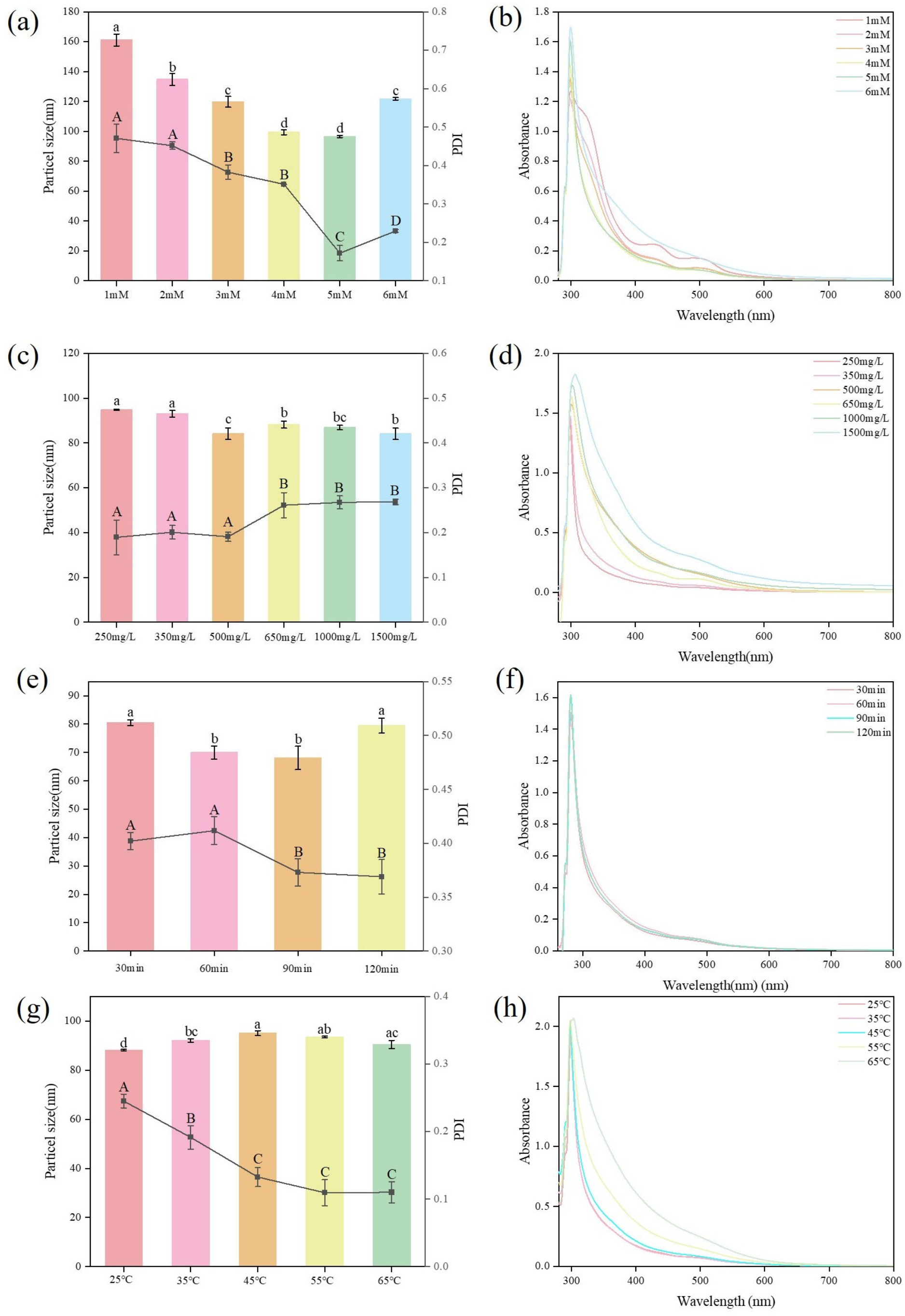

Supplement: Supplementary file 1 [file foods-14-03002-s001.zip › foods-3802764-supplementary.pdf]
